# Application of high-throughput amplicon sequencing-based SSR genotyping in genetic background screening

**DOI:** 10.1186/s12864-019-5800-4

**Published:** 2019-06-03

**Authors:** Tiantian Li, Zhiwei Fang, Hai Peng, Junfei Zhou, Pengcheng Liu, Yanyan Wang, Wenhui Zhu, Lun Li, Quanfang Zhang, Lihong Chen, Lili Li, Zhihao Liu, Weixiong Zhang, Wenxue Zhai, Long Lu, Lifen Gao

**Affiliations:** 1 Institute for Systems Biology, Jianghan University, Wuhan, 430056 Hubei China; 2 Institute of Genetics and Developmental Biology, Chinese Academy of Sciences, Beijing, 100101 China; 3 Bio-Tech Research Center, Shandong Academy of Agricultural Sciences/Shandong Provincial Key Laboratory of Crop Genetic Improvement, Ecology and Physiology, Jinan, 250100 China; 4 Division of Biomedical Informatics, Cincinnati Children’s Hospital Research Foundation, 3333 Burnet Avenue, Cincinnati, OH 45229-3026 USA

**Keywords:** SSR-based genetic background screening, *Xa21*, Marker-assisted backcrossing, Transgenesis, CRISPR/Cas9

## Abstract

**Background:**

Host genetic backgrounds affect gene functions. The genetic backgrounds of genetically engineered organisms must be identified to confirm their genetic backgrounds identity with those of recipients. Marker-assisted backcrossing (MAB), transgenesis and clustered regularly interspaced short palindromic repeats (CRISPR)/CRISPR-associated protein 9 (CRISPR/Cas9) editing are three commonly used genetic engineering techniques. However, methods for genetic background screening between genetically engineered organisms and corresponding recipients suffer from low efficiency, low accuracy or high cost.

**Results:**

Here, we improved our previously reported AmpSeq-SSR method, an amplicon sequencing-based simple sequence repeat (SSR) genotyping method, by selecting SSR loci with high polymorphism among varieties. Ultimately, a set of 396 SSRs was generated and applied to evaluate the genetic backgrounds identity between rice lines developed through MAB, transgenesis, and CRISPR/Cas9 editing and the respective recipient rice. We discovered that the percentage of different SSRs between the MAB-developed rice line and its recipient was as high as 23.5%. In contrast, only 0.8% of SSRs were different between the CRISPR/Cas9-system-mediated rice line and its recipient, while no SSRs showed different genotypes between the transgenic rice line and its recipient. Furthermore, most differential SSRs induced by MAB technology were located in non-coding regions (62.9%), followed by untranslated regions (21.0%) and coding regions (16.1%). Trinucleotide repeats were the most prevalent type of altered SSR. Most importantly, all altered SSRs located in coding regions were trinucleotide repeats.

**Conclusions:**

This method is not only useful for the background evaluation of genetic resources but also expands our understanding of the unintended effects of different genetic engineering techniques. While the work we present focused on rice, this method can be readily extended to other organisms.

**Electronic supplementary material:**

The online version of this article (10.1186/s12864-019-5800-4) contains supplementary material, which is available to authorized users.

## Background

The expression of a particular gene is influenced by expression changes in other interacting genes in the gene network, that is, the genetic background in which the gene is located. For example, the disease resistance provided by the rice bacterial blight resistance gene *Xa3*/*Xa26* or the three-gene pyramid (*xa5* + *xa13* + *Xa21*) is influenced by host background [1, 2]. The function of the rice blast resistance gene *pi21* is influenced by a closely linked gene conferring poor flavour [3]. The brown planthopper resistance gene *Bph6* in *indica* rice shows a quicker and stronger effect toward brown planthopper than that in *japonica* rice [4].

Near-isogenic lines (NILs) are ideal materials for quantitative trait locus mapping, map-based gene isolation and gene functional analysis [5–7]. Marker-assisted backcrossing (MAB), transgenesis and clustered regularly interspaced short palindromic repeats (CRISPR)/CRISPR- associated protein 9 (CRISPR/Cas9) editing are widely used methods to develop NILs. The basis of MAB is the transfer of a specific allele from a donor line to a recipient line through artificial hybridization. Multigeneration backcrossing is necessary to select against donor introgression and linkage drag, and a large number of individual plants is required to obtain the desired genotypes and phenotypes [5–7]. Despite being time-consuming and laborious, MAB is very useful and is indispensable for target genes that have not been cloned. For genes that have been cloned, MAB appears to be less efficient than transgenic technology, which can transfer only the target gene into the host genome and therefore avoid the problem of linkage drag. For example, rice varieties with high vitamin A content, high resistance to stresses, or high cellulose content have been rapidly generated through this technology [8–10]. It has been reported that transgenesis has a lower impact on the whole-genome expression profile than MAB [11, 12]. An unavoidable problem associated with transgenic technology is the random integration of the target gene in the host genome. Fortunately, the recently developed CRISPR/Cas9 gene editing technology seems to be an ideal solution. This technology can edit the target gene specifically without changing the natural location of the target gene [13, 14]. The CRISPR/Cas9 system allows editing of several bases or even a single base within a target gene. Recently, an herbicide-resistant rice variety was developed by editing a single base [15]. Due to its precision, high efficiency, and genome-scale gene editing capability [16], CRISPR/Cas9-mediated gene editing technology has been rapidly developed and is widely used in functional studies and crop improvement [17–19].

Regardless of which methods are used, the ultimate goal is to create NILs that have consistent genetic backgrounds with those of recipients. Whole-genome re-sequencing [20], electrophoresis-based single sequence repeat (SSR) genotyping [4] and high-throughput single nucleotide polymorphism (SNP) genotyping [21] have been reported to be useful for detecting the genetic background of NILs. SSRs are widely distributed with high density and high variability in eukaryotic genomes. In addition, SSRs have other advantages, such as co-dominance, sequence diversity and high conservation of their flanking regions, making SSRs ideal genetic markers. SSRs have been widely used for DNA fingerprinting, genetic diversity analysis, and variety identification [22–24]. Widely used detection techniques for SSR markers include a variety of electrophoresis techniques, including agarose, polyacrylamide gel electrophoresis, and capillary electrophoresis (CE). These electrophoresis techniques can detect differences in length but cannot detect base variations that do not cause changes in length. Moreover, if the difference in length is not obvious, the resolution of electrophoresis techniques is also very limited. For example, CE, which is currently the most sensitive electrophoresis technique, can detect one base-pair (bp) length differences under certain experimental conditions [25], but is more reliable for detection of length changes *>* 5 bp [26]. More importantly, when the number of SSR loci to be detected is large, electrophoresis- based SSR detection techniques are time-consuming and labour-intensive. Therefore, gel electrophoresis has been used to analyse only a few SSR loci, such as 27, 47, and 48 SSR loci in cabbage [24], barley (*Hordeum vulgare* L.) [22], and rice (National Agricultural Standard of China No. NY/T 1433–3014), respectively. Sequencing-based high- throughput methods are significantly more time-efficient and less laborious than conventional electrophoresis-based genotyping approaches. In our previous study, we developed an accurate and efficient SSR genotyping method, named AmpSeq-SSR, by combining multiplexing polymerase chain reaction (PCR), amplicon sequencing and bioinformatics analysis. Through selection of SSR loci with high polymorphism between samples, we further improved AmpSeq-SSR and used it to identify the genetic backgrounds of rice lines developed by MAB, transgenesis, CRISPR/Cas9 editing, and their respective recipient varieties in this study. *Xa21*, the first cloned bacterial blight (BB) resistance gene, was selected as the target gene [27]. Due to its broad spectrum and high level of resistance it confers against rice BB pathogens, *Xa21* has been widely introduced into susceptible rice varieties by transgenesis or MAB technology [28–31], and the molecular mechanism of *Xa21*-mediated resistance is a popular research topic in the field of plant disease resistance [1, 29, 32, 33].

In our previous study, an *Xa21* transgenic rice variety [28] and an *Xa21* MAB-produced rice variety [12] were developed by introducing *Xa21* into the recipient rice line D62B. To evaluate and compare the genetic variations introduced by MAB, transgenesis and CRISPR/Cas9 techniques, an artificial mutant of the *Xa21* gene was first developed by editing the *Xa21* gene in *Xa21* transgenic rice through CRISPR/Cas9 technology, and then sequencing-based high-throughput SSR genotyping was carried out on *Xa21* transgenic rice, *Xa21* MAB-produced rice, and *Xa21* gene editing mutants and their respective recipients. Through comparisons with the corresponding recipient lines, the genetic background identity between rice materials developed by MAB, transgenesis, and CRISPR/Cas9 techniques and recipient rice was determined. This genome-wide SSR genotyping method accurately assessed, for the first time, the genetic variations introduced by existing genetic engineering methods, and our results provide insight for the study and application of functional genes.

## Results

### Generation of *Xa21* mutant lines

**Fig. 1:**
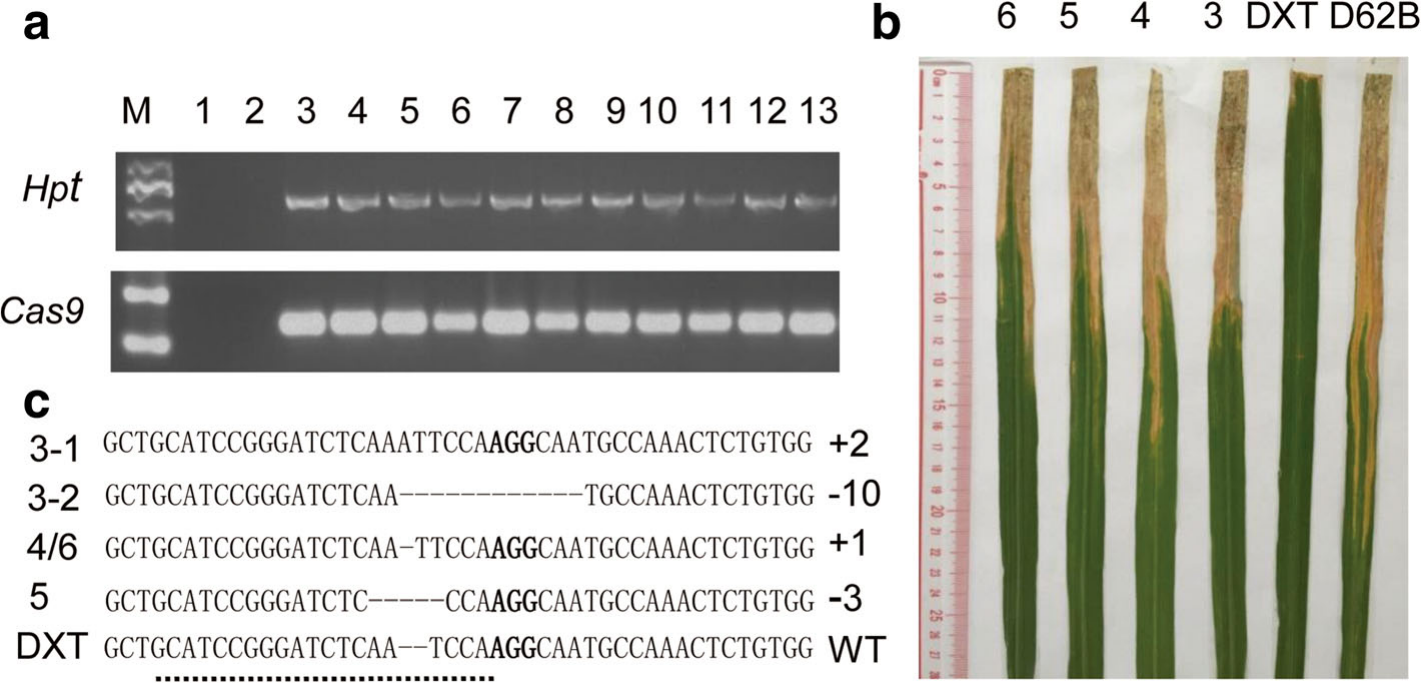
Identification of *Xa21* mutant lines in the T_0_ generation. **a** PCR-detected presence and absence of individual transgenes (*Hpt* and *Cas9*) in T_0_ transgenic plants; 1–2 were T_0_ transgenic-negative lines, and 3–13 were T_0_ transgenic-positive lines. **b** Susceptible phenotype of T_0_ transgenic lines 3 to 6. **c** Sequence alignment of the sgRNA target region showing the altered bases in T_0_ transgenic lines 3 to 6. + 2: 2 bp insertion. -10, 10 bp deletion; + 1, 1 bp insertion; − 3, 3 bp deletion; WT, *Xa21* wild type. The sgRNA-targeted site is denoted with a dotted line, and the PAM sequence is in black bold font

In the T_0_ generation, 13 independent transgenic lines were obtained, and 11 were transgenic-positive lines that carried the marker gene *Hpt* and the *Cas9* gene (Fig. 1a). Inoculation with the *Xoo* P6 strain at the peak tillering stage showed that four lines (lines 3~6) among the 11 transgenic-positive T_0_ lines showed susceptible phenotypes (Fig. 1b). Because *Xa21* is a dominant resistance gene, *Xa21* mutation in these susceptible T_0_ plants should be homozygous or compound heterozygous, while that in the resistant T_0_ transgenic lines should be heterozygous or non-existent. We investigated the resistance of the T_1_ progeny plants of the 11 T_0_ transgenic-positive lines and found that all the T_1_ plants of the four susceptible T_0_ lines (lines 3~6) were susceptible, five T_1_ lines (lines 7~11) were mixed, and two T_1_ lines (line 12 and 13) were resistant, confirming the homozygous/compound heterozygous and heterozygous mutation patterns of the corresponding T_0_ plants (Table 1). Analysis of the PCR products from the targeted sites revealed that line 3 was compound heterozygous and harboured di-allelic (2 bp insertion/10 bp deletion) mutations in the *Xa21* gene, line 4 and line 6 were homozygous for a 1-bp insertion, and line 5 was homozygous for a 3-bp deletion in the *Xa21* gene (Fig. 1c, Additional file 1: Figure S1). This result showed that the CRISPR/Cas9 system can produce specific and homozygous targeted gene editing in one generation in rice, which is consistent with a previous report [13]. The sequences of the PCR primers are listed in Additional file 2: Table S1.

**Table 1:** Phenotypes and mutation patterns of the 11 T_0_ transgenic-positive lines

T_0_ line	T_0_ generation				T_1_ segregation ratio		
	T-DNA	Phenotype	Zygosity	Genotype	R	S	Total
3	+	S	Com-He	-10, + 2	0	30	30
4	+	S	Ho	+ 1, + 1	0	30	30
5	+	S	Ho	-3, −3	0	20	20
6	+	S	Ho	+ 1, + 1	0	20	20
7	+	R	He	–	3	17	20
8	+	R	He	–	10	10	20
9	+	R	He	–	7	12	19
10	+	R	He	–	7	33	40
11	+	R	He	–	10	15	25

*R* resistant, *S* susceptible, *Ho* homozygote, *He* Heterozygote, *Com-he* compound heterozygote. -10, 10-bp deletion; + 2, 2-bp insertion; + 1, 1-bp insertion; −3, 3-bp deletion; −, genotype not tested

Detailed analysis was performed on the 30 T_1_ plants of line 3. Fifteen of the 30 T_1_ plants were either homozygous for a 10-bp deletion or 2-bp insertion, and the rest were heterozygous for di-allelic mutations. The lines with 2-bp insertions and 10-bp deletions were named line 3–1 and line 3–2, respectively. Moreover, line 3–1 and line 3–2 had four plants (26.7%) that were transgene clean (*Hpt*- and *Cas9*-free) and homozygous for mutated *Xa21*, respectively, according to the results of *Hpt* and *Cas9* PCR detection. These results indicated that transgene-clean and homozygous mutants could be acquired with high efficiency through the CRISPR/Cas9 system.

**Fig. 2:**
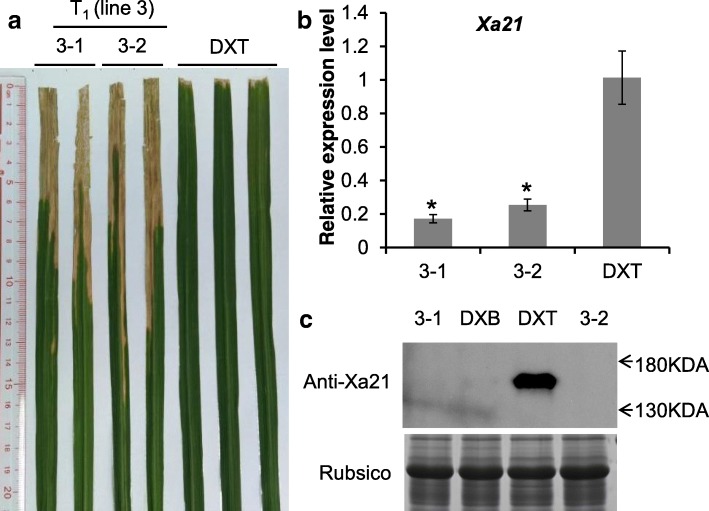
Resistance performance and expression of *Xa21* in mutants**. a** Susceptible phenotype of the T_1_ plants. **b** Expression levels of *Xa21* in T_1_ plants and wild-type DXT as detected by qPCR. Asterisks indicate a significant difference compared to the DXT line at *P* < 0.05 with Student’s *t-test*. **c** XA21 protein levels in T_1_ plants and wild-type plants as detected by western blot analysis. 3–1 and 3–2, *Xa21* mutant lines (T_1_ plants of line 3); DXT, wild-type line (*Xa21* transgenic line); DXB, *Xa21* MAB-produced line; rubisco, the control for total leaf protein

The susceptible phenotype of line 3 was stably inherited in the T_1_ generation (Fig. 2a). The expression levels of *Xa21* in the transgene-clean and homozygous mutants from lines 3–1 and 3–2 (the T_1_ plants of line 3) were revealed to be decreased significantly by quantitative PCR analysis (Fig. 2b). XA21 protein was detected distinctly in DXT, while no signal was detected in line 3–1, line 3–2, or the *Xa21* MAB-produced line DXB, suggesting that the translation of XA21 was totally suppressed in the mutants or was below the detection limit of the XA21 antibody (Fig. 2c). The transgene-clean and homozygous *Xa21* mutants in line 3–1 were named Xa21m.

### Summary of the SSR genotyping data

**Fig. 3:**
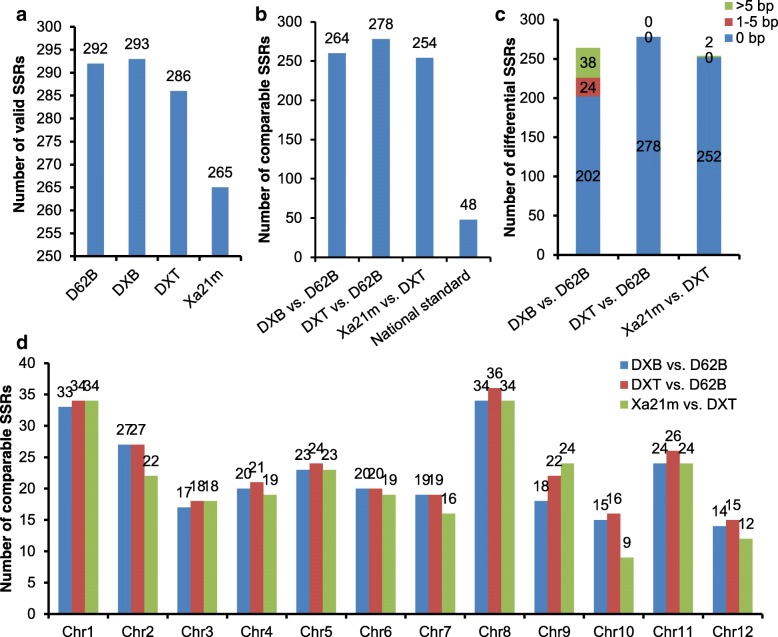
SSR genotyping of four rice samples. **a** Valid SSRs identified in D62B, DXB, DXT and Xa21m. **b** Comparable SSRs identified between two samples. **c** Length differences of differential SSRs identified between two samples; > 5, 1–5 and 0 bp refer to the amplicon length differences between two samples, which are distinguishable, hardly distinguishable and indistinguishable in electropherograms, respectively. **d** Distribution of the comparable SSRs on the 12 chromosomes of rice between two samples

For evaluation of the genetic backgrounds of the rice materials developed by the three techniques, the genotypes of the 396 target SSRs in D62B, DXB, DXT and Xa21m were identified by the AmpSeq-SSR genotyping method. The sequences of primers used to amplify the target SSRs are listed in Additional file 3: Table S2. A total of 366 SSR loci were detected in the four samples, suggesting the high amplification efficiency of the mixed PCR primers (Additional file 4: Table S3). The numbers of valid SSRs (see Methods) in D62B, DXB, DXT and Xa21m were 292, 293, 286 and 265, respectively (Fig. 3a; Additional file 4: Table S3, Additional file 5: Table S4, Additional file 6: Table S5 and Additional file 7: Table S6). The numbers of comparable SSRs (see Methods) in the DXB vs. D62B, DXT vs. D62B and Xa21m vs. DXT comparisons were 264, 278 and 254, respectively, and the average number of comparable SSRs in the three pairs was 265.3 (Fig. 3b; Additional files 5, 6 and 7: Tables S4, S5 and S6), far more than the 48 SSRs used for rice identification (National Agricultural Standard of China No. NY/T 1433–3014). The number of comparable SSRs on each of the 12 chromosomes of rice ranged from 9 to 36, showing the high genome coverage of the detected SSRs (Fig. 3d).

### High genomic variations introduced by the MAB method

Among the 264, 278 and 254 comparable SSRs (see Methods) between DXB and D62B, DXT and D62B, and Xa21m and DXT, 62 (23.5%), 0 (0.0%) and 2 (0.8%) of the SSRs had different genotypes, respectively, indicating the high genomic variation induced by the MAB method (Fig. 3c; Additional files 5, 6 and 7: Tables S4, S5 and S6). Approximately 38.7% (24/62) of the differential SSRs between DXB and D62B had differences in amplicon length less than 5 bp, which could hardly be distinguished in electropherograms (Fig. 3c). Of the 48 SSRs for rice identification adopted by the National Agricultural Standard of China (Standard No. NY/T 1433–3014), 41 SSRs were detected effectively in both DXB and D62B, and eight SSRs (19.5%) showed different genotypes between the two samples (Table 2). However, only four of the eight differential SSRs varied in length by more than 5 bp and could thus be discriminated by high-concentration gel electrophoresis (Table 2). The results showed the high accuracy of AmpSeq-SSR for plant genetic background screening.

**Table 2:** Genotypes of the differential SSRs between D62B and DXB included in the National Agricultural Standard of China

Amplicon	D62B			DXB			Name in national standard
	Genotype	Coverage	Stutter ratio	Genotype	Coverage	Stutter ratio	
AMPL1563384	TC10	47	0.13	TC9	19	0.32	RM561
AMPL1563300	TCA16	45	0.16	TCA15	20	0.15	RM19
AMPL1563868	CA6	88	0.40	CA5	77	0.31	RM316
AMPL1563825	AAG8	265	0.08	AAG11	104	0.13	RM339
AMPL1563618	GCA8	247	0.15	GCA9	179	0.40	RM598
AMPL1563371	ATG18	198	0.31	ATG15	189	0.20	RM424
AMPL1563368	AAT6	481	0.07	AAT4_AAT10	284	0.08	RM71
AMPL1563350	GAA15	192	0.11	GAA8	694	0.06	RM423

The coverage represents the number of reads that support the genotype. The stutter ratio represents the ratio of the numbers of reads for the minor and major genotypes

**Fig. 4:**
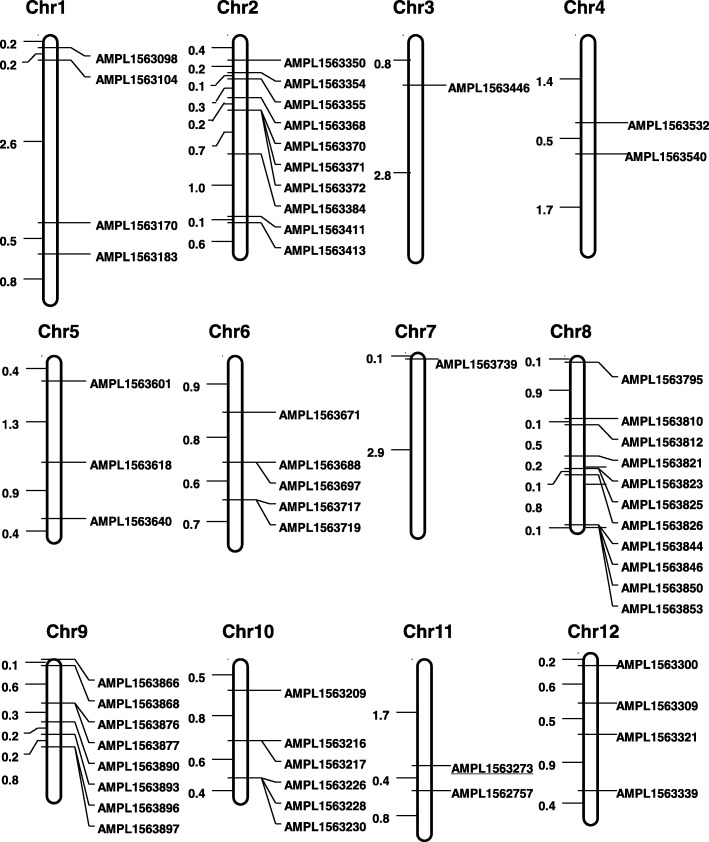
Distribution of the amplicons with differential SSRs. Amplicons with underscores represent SSRs exhibiting different genotypes between DXT and Xa21m, and amplicons without underscores represent SSRs exhibiting different genotypes between DXB and D62B. The number to the left of the chromosome map, multiplied by 10^7^ bp, represents the physical distance between two adjacent amplicons

Among the 254 comparable SSRs between Xa21m and DXT, only two SSRs in amplicon AMPL1563273 that were located on chromosome 11 exhibited different genotypes (Fig. 4, Additional file 7: Table S6). The percentages of different SSRs between DXT and D62B (0.0%) and between Xa21m and DXT (0.8%) were clearly far less than the percentage of different SSRs between DXB and D62B (23.5%) (Fig. 3c).

### Chromosome distribution of the differential SSRs between DXB and D62B

The 62 differential SSRs between DXB and D62B were distributed across the 12 chromosomes of rice, especially on chromosomes 2, 8, 9 and 10 (12, 13, 8, and 6 SSRs, respectively) (Fig. 4). Specifically, 30 (48.4%) SSRs were distributed in the intergenic regions, and 32 (51.6%) were distributed in the intragenic regions. In addition, 39 (62.9%) of the differential SSRs were located in non-coding regions, 13 (21.0%) were in untranslated regions, and only 10 (16.1%) were in coding regions (CDS). The most abundant types of differential SSRs were trinucleotide repeats (51.6%). Most importantly, all of the SSR variants in the CDS were frameshift-free trinucleotide repeats (Additional file 5: Table S4). Using the criteria of at least a two-fold expression change and a false discovery rate (FDR) no greater than 0.01, we found that of the 30 genes with differential SSRs, four (LOC_Os01g60920, LOC_Os04g31110, LOC_Os02g15594, and LOC_Os07g02280) showed significant differential expression between D62B and DXB based on the whole-genome expression profiles in our previous report [12]. The expression patterns of two genes (LOC_Os07g02280 and LOC_Os01g60920) were validated by qPCR (Additional file 1: Figure S2). The primers are listed in Additional file 2: Table S1.

## Discussion

### Advantages and disadvantages of the AmpSeq-SSR method in genetic background screening

The most important factor in cloning QTLs or genes using NILs is to determine the extent to which NILs recover the background of the recurrent parent. The AmpSeq-SSR method has comparative advantages over existing methods for genetic background identification. First, the multiplex PCR-based AmpSeq-SSR method only requires a small amount of template DNA, e.g., 10 ng [26]. Second, 396 target SSRs of four samples were detected at once, whereas the whole process of detecting 254 SSR markers by PCR amplification and agarose gel electrophoresis took more than three years [20]. Because of the existence of a 384-well barcode, we can actually analyse 768 samples at once by sequencing two chips. Third, AmpSeq-SSR is compatible with existing rice identification systems. Forty-seven of the 48 SSRs used for rice identification (National Agricultural Standard of China No. NY/T 1433–3014) were included in the 396 target SSRs. More importantly, 42, 41, 42, and 41 SSRs, respectively, of the 48 SSRs were detected in D62B, DXB, DXT, and Xa21m (Additional file 4: Table S3). Fourth, AmpSeq-SSR provides more accurate genotyping results than electrophoresis and whole-genome sequencing (WGS)-based SSR detection techniques [26].

PCR [34] and sequencing errors [35] affected the genotyping accuracy of AmpSeq-SSR, and these errors also plague WGS-based SSR genotyping techniques. However, Ampseq-SSR can provide reliable genotyping results by adjusting the sequence coverage and the stutter ratio of the genotyping system. When the SSR stutter ratio was set to less than 0.5, the accuracy of AmpSeq-SSR genotyping with the coverage of 10× and 50× was 99.73 and 99.87%, respectively [26].

### The extended application of AmpSeq-SSR genotyping-based background screening

The traditional method of gene cloning involves first screening the parents and F_2_ mutants with a small number of molecular markers distributed on each chromosome and comparing the PCR product bands of the three samples in parallel electrophoresis to preliminarily map the target gene to the chromosome; then, upon screening a large number of F_2_ individuals, new molecular markers are designed for chromosome walking to obtain the precise chromosome location of the target gene. The entire process requires extensive PCR amplification and parallel electrophoresis. The AmpSeq-SSR method can detect 396 SSR loci in 768 samples at once with the help of 384 barcodes. In this and our previous study, one differential SSR in amplicon AMPL1562757 with different genotypes between *Xa21* MAB-produced rice lines and recipient lines happened to be located near *Xa21* [26]. In addition, AmpSeq-SSR can effectively assist in the selection of individual with the closest genetic background to the recipient for next-generation backcrossing. In short, the method we present can be extended to gene mapping and speed up the process of MAB.

### Unintended effects of genomic variations on recipient phenotypes

Previous reports have shown that SSR variations located within the 3′ untranslated region (UTR), 5′ UTR, introns, or CDS of a gene affect gene function [36–40]. We are concerned about the unintended effects of the 62 differential SSRs on the traits of D62B and DXB. Of the 62 differential SSRs between D62B and DXB, nine were located in the intron regions, 30 were in the intergenic regions, 13 were in the 5′ or 3′ UTR, and only 10 were in the CDS (Additional file 5: Table S4). However, all of the SSR variants in the CDS were frameshift-free trinucleotide repeats and therefore may not affect the functions of the genes (Additional file 5: Table S4). Analysis of the expression of the 30 genes with differential SSRs revealed that four genes showed significantly differential expression between D62B and DXB (Additional file 1: Table S1 in [12]). We compared the main agronomic traits, such as lesion length, plant height, tiller numbers, panicle length, seeding ratio, and 1000 grain weight and found that D62B and DXB had no significant differences in the main agronomic traits except BB resistance [12]. The large genetic difference between D62B and DXB was probably due to the fact that we only selected individuals that were similar in appearance to the recipient line D62B and were resistant to *Xoo* to backcross with D62B without any molecular marker-based background selection during the first five generations of backcrossing. With the aid of positive selection for target genes and negative selection for background, the recipient genome can be recovered over 99% during the MAB process [7].

## Conclusions

This study provided a high-throughput SSR genotyping-based genetic background screening method and used the method to screen the genomic backgrounds of rice materials developed by commonly used genetic improvement techniques, including MAB, transgenesis, and CRISPR/Cas9 techniques. We found that a large proportion of SSRs showed different genotypes between MAB-produced rice and recipient rice, whereas SSRs between transgenic rice and CRISPR/Cas9-mediated rice and their respective recipients differed only slightly. Furthermore, most differential SSRs introduced by MAB technology were located in non-coding regions, and all of the differential SSRs located in the coding regions were frameshift-free trinucleotide repeats. This method is not only useful for the evaluation of genetic resources but also expands our understanding of the unintended effects introduced by different genetic improvement techniques. While the work we present focused on rice, this method can be readily extended to other organisms.

## Methods

### Rice plants

D62B is an *indica* rice that is widely used as a parental line for hybrid rice in China. *Xa21* was the first cloned bacterial blight-resistant gene from *Oryza longistaminata* [27]. The *Xa21* transgenic rice line DXT was generated by transferring *Xa21* into D62B through transgenic technology [28] and the *Xa21* MAB-produced rice line DXB was bred by introgressing *Xa21* into D62B through MAB technology using the rice variety IRBB21 as the donor [12]. IRBB21 was constructed by backcrossing the wild African species *O. longistaminata* containing *Xa21* (*Xa21* donor parent) five times to the recurrent parent *O. sativa* (IR24) [41]. The T_12_ generation of DXT and the BC_6_F_2_ generation of DXB with homozygous *Xa21* and stable agronomic traits were used for analysis in this study. Xa21m, a *Xa21* mutant line, was developed using DXT as a recipient through CRISPR/Cas9 technology in this study (Additional file 1: Figure S3). These three pairs of rice materials, including DXB vs. D62B, DXT vs. D62B and Xa21m vs. DXT, constitute a relatively ideal system to screen the genetic backgrounds of rice lines developed by transgenesis, MAB, and CRISPR/Cas9 techniques. The use and preservation of the genetically modified materials were subject to the Cartagena Protocol on Biosafety.

### *Xanthomonas oryzae* pv. *oryzae* (*Xoo*) cultivation and rice infection

Bacterial blight in rice is caused by *Xoo*. The *Xoo* P6 strain, a common strain used to identify *Xa21* function, is from the Philippines. The preserved P6 strain was revived in potato sugar agar (PSA) medium (potato, 300 g/L; sugar, 15 g/L; Na_2_HPO_4_•12H_2_O, 2.0 g/L; Ca (NO3)_2_•4H_2_O, 0.5 g/L; agar, 15 g/L) at 28 °C for three days and then inoculated onto fresh PSA medium. Two days later, the activated P6 strain was resuspended with sterile water, and the concentration was adjusted to 10^9^ cells/ml. The rice plants were infected with P6 at the peak tillering stage using the leaf-clipping method [27]. Photographs of infected leaves in each rice line were taken 12 days after inoculation.

### Construction of the CRISPR/Cas9 vector

Bases 1852~1871 of the *Xa21* gene coding region were selected to form a small guide RNA (sgRNA) with a sequence of GCATCCGGGATCTCAATCCA. The source and construction of the CRISPR/Cas9 vector was based on a previous report [42]. Briefly, the forward primer CRP-Xa21F and the reverse primer CRP-Xa21R were designed according to the sequence of *Xa21* sgRNA. Then, the *Xa21* sgRNA was introduced into the U6 expression cassette through three rounds of PCR. The first round of PCR was performed by using the plasmid pCXUN-Cas9/U6 containing the U6 expression cassette as a template, the forward primer CRP-Xa21F and the reverse primer U6-R. The second round of PCR was performed with the CRP-Xa21R and U6-F primers using pCXUN-Cas9/U6 as the template. Finally, the third round of PCR was carried out by using the mixture of the first two rounds of PCR products as template and U6-F and U6-R as primers to obtain the U6 promoter-driven sgRNA expression cassette. Then, the expression vector pCXUN-Cas9/Xa21 was obtained by ligating the U6 promoter-driven sgRNA expression cassette into the *Kpn*I-digested vector backbone pCXUN-Cas9 through a recombination reaction. The primers are listed in Additional file 2: Table S1.

### *Agrobacterium*-mediated rice transformation

The constructed vector pCXUN-Cas9/Xa21 was transferred into the *Agrobacterium tumefaciens* strain EHA105 by the heat shock method. The mature embryo-derived callus cells of DXT were used for *Agrobacterium*-mediated rice transformation according to a method described by [30].

### Detection of *Xa21* mutants

Genomic DNA of transgenic plants was extracted using the hexadecyltrimethylammonium bromide (CTAB) method and further used for PCR amplification with specific primers. Hpt-F/R primers for detection of the marker gene hygromycin were used to identify transgenic-positive plants. Cas-Xa21F/R primers were designed to amplify the genomic regions surrounding on- and off-target sites. The PCR products of the Cas-Xa21F/R primers were directly subjected to Sanger sequencing using the Cas-Xa21F primer or were cloned into the pEASY-T vector and then Sanger-sequenced using M13F primers to determine the gene editing sites. The primers are listed in Additional file 2: Table S1.

### Selection of target SSR loci for genetic background screening

Based on the sequencing data of 3105 SSR loci of eight rice varieties in our previous report [26], we improved AmpSeq-SSR in this study by selecting SSRs with high polymorphism among varieties from the 3105 SSR loci to form a target SSR locus library for genetic background screening. All of the sequenced reads were first aligned with the *japonica* reference genome (irgsp1.0) [43] with Bowtie 2 (version 2.1.0) [44], and then a Perl script was used to extract the genotype at an SSR locus based on the alignment information. The allele with the most reads supported at individual SSR loci was designated as the major allele of the SSR locus, and those with the second-largest number of reads were designated as minor allele. The ratio between the read numbers for the minor and major alleles was taken as the stutter ratio of the SSR locus, and the major allele was recorded as the genotype of an SSR locus because only a homozygous allele was expected for an SSR locus in inbred rice lines. Each SSR genotype is presented as the motif followed by the number of repeats, e.g., AT10. Two SSRs separated by a distance of at least 10 bp were considered to be two different SSRs, while those separated by less than 10 bp were considered to be one SSR (the genotype of this type of SSR is presented as the genotype of two SSRs plus one -, e.g. AAT4_AAT10). The detailed steps of sequence read processing and SSR genotype calling were performed as described in our previous report [26]. We collected valid SSR loci from each variety with the following criteria: coverage by at least 20 reads and a stutter ratio lower than 0.5. The genotypes of the valid SSR loci in eight varieties were then compared with each other to obtain the differential SSR loci. The number of variety pairs that could be distinguished by one SSR locus divided by 28 (the number of total possible pairs of the eight varieties) was taken as the diversity index of the SSR locus. An SSR locus with a diversity index greater than 0.3 that was less than one megabase pair away from nearby SSR loci was defined as a target SSR locus. Ultimately, a total of 396 SSRs, including the 48 SSRs currently used for rice identification by the National Agricultural Standard of China (Standard No. NY/T 1433–3014), were used for genetic background screening. In detail, 23 to 50 SSRs were distributed on each chromosome of the rice genome (Additional file 3: Table S2).

Primers for amplification of the 396 SSRs were designed by Thermo Fisher Company at https://ampliseq.com/ and then synthesized as a primer pool. Then, the 396 SSR loci were amplified by 256 pairs of primers in 16 cycles according to the procedures provided in the instructions. The primer sequences are listed in Additional file 3: Table S2. The amplification products were digested with FuPa reagent to remove redundant primer sequences, and then barcode sequences were incorporated to differentiate different samples. AmpliSeq library construction was carried out using an Ion AmpliSeq Library Kit 2 (4,475,345 Thermo Fisher Scientific, Waltham, MA, USA) according to the manufacturer’s instructions. The constructed AmpSeq-SSR library was quantified by the TaqMan probe method and then mixed in equimolar amounts for sequencing on an Ion S5 sequencer (A27212, Thermo Fisher Scientific, Waltham, MA, USA) by single-end sequencing with a 300 bp read length.

### Differential SSR identification

Based on the results of SSR genotyping, we first called the valid SSR loci in each sample (those that were covered by at least 10 reads and had a stutter ratio lower than 0.5). When an SSR was valid in two samples, the SSR was recorded as a comparable SSR between the two samples. The genomic variations introduced by the different genetic engineering techniques were determined by comparing the genotypes of the comparable SSR loci of the genetically engineered organisms with those of the corresponding recipients.

### Quantitative reverse-transcription PCR (qPCR)

Total RNA from rice leaves was extracted using Invitrogen TRIzol reagent (Invitrogen, Thermo Fisher). DNase I-treated RNA was used for first-strand cDNA synthesis using M-MLV Reverse Transcriptase (Promega) and oligo (dT)18 primers according to the manufacturer’s protocol in a 20-μl reaction volume. Specific pairs of primers for SYBR Green detection and quantification of target genes were designed using the web tool Primer-BLAST provided by the National Center for Biotechnology Information. The primer sequences are listed in Additional file 2: Table S1. qPCR was performed using TransStart® TipTop Green qPCR SuperMix reagent (Cat. No. AQ141, TransGen Biotech, China) on an Applied Biosystems StepOnePlus™ Real-Time PCR System. The expression level of the *Xa21* gene in *Xa21* mutants was calculated by using DXT as the calibrator to normalize the relative expression. D62B was used as a reference sample to calculate the expression levels of the LOC_Os01g60920 and LOC_Os07g02280 genes in DXB. A reaction with the endogenous *ubiquitin* gene was run in parallel as a control reaction [45]. Triplicate samples for each tested line were prepared for real-time PCR assays. The 2^−ΔΔ*C*^_T_ method was used to calculate relative changes in gene expression [46]. Student’s *t-test* was used to test the significance of each difference in expression levels between two rice lines with a cut-off of a *p* value less than 0.01.

## Additional files

Additional file 1: **Figure S1.** The sequencing figure shows the mutation patterns of the T_0_ transgenic lines 3 to 6. + 2, 2 bp insertion; − 10, 10 bp deletion; + 1, 1 bp insertion; − 3, 3 bp deletion. WT, *Xa21* wild type; _ indicates inserted bases, and arrows indicate deletion start sites. **Figure S2.** Expression validation of the genes in which the differential SSRs were distributed. D62B was used as a reference sample, and the rice *ubiquitin* gene was used as an endogenous control. **Figure S3.** Development of DXB, DXT and Xa21m. *O. longistaminata*: *Oryza longistaminata*, *Xa21* donor parent; IR24: *Oryza sativa*, recurrent parent. MAB: marker-assisted backcrossing. IRBB21, *Xa21* donor parent for DXB. D62B, the recipient rice for DXB and DXT; DXB, *Xa21* MAB-produced rice; DXT, *Xa21* transgenic rice; Xa21m, CRISPR/Cas9-mediated *Xa21* mutant rice. (PPTX 98 kb)

Additional file 2: **Table S1** Primers for vector construction, *Xa21* detection and gene expression analysis. (XLSX 12 kb)

Additional file 3: **Table S2** Primers for the detection of 396 SSR loci. (XLSX 35 kb)

Additional file 4: **Table S3** Total SSRs detected in D62B, DXB, DXT and Xa21m. The genotype is the major genotype of the SSR locus, represented as the repeat unit with the repeat number, e.g., CGC4. The coverage represents the number of reads that supported the genotype. The stutter ratio represents the ratio of read numbers of the minor and major genotypes. The name in the national standard represents the name of the differential SSR in the National Agricultural Standard of China No. NY/T 1433–3014. (XLSX 45 kb)

Additional file 5: **Table S4** Valid and comparable SSRs in DXB and D62B. The valid SSR loci in each sample are those SSR loci covered by at least 10 reads and with stutter ratios lower than 0.5. Comparable SSRs between two samples are those SSR loci that are valid in both samples. The genotype is the major genotype of the SSR locus, represented as the repeat unit with the repeat number, e.g., CGC4. The coverage represents the number of reads that support the genotype. The stutter ratio represents the ratio of the read numbers of the minor and major genotypes. Asterisks represent the differential SSRs between two samples; > 5, 0 and 1–5 represent the length differences of differential SSRs between two samples. SSRs with length differences less than 5 bp were hardly distinguished by electrophoresis. The name in the national standard represents the name of the differential SSR in the National Agricultural Standard of China No. NY/T 1433–3014. The MSU ID shows the gene symbols of the genes in which the different SSRs are distributed. The region of genes represents the region of the differential SSR within the gene, e.g., the UTR. (XLSX 40 kb)

Additional file 6: **Table S5** Valid and comparable SSRs in DXT and DXB. The valid SSR loci in each sample are those SSR loci covered by at least 10 reads and with stutter ratios lower than 0.5. Comparable SSRs between two samples are those SSR loci that are valid in both samples. The genotype is the major genotype of the SSR locus, represented as the repeat unit with the repeat number, e.g., CGC4. The coverage represents the number of reads that support the genotype. The stutter ratio represents the ratio of the read numbers of the minor and major genotypes. (XLSX 37 kb)

Additional file 7: **Table S6** Valid and comparable SSRs in DXT and Xa21m**.** The valid SSR loci in each sample are those SSR loci covered by at least 10 reads and with stutter ratios lower than 0.5. The comparable SSRs between two samples are those SSR loci that are valid in both samples. The genotype is the major genotype of the SSR locus, represented as the repeat unit with the repeat number, e.g., CGC4. The coverage represents the number of reads that support the genotype. The stutter ratio represents the ratio of the read numbers of the minor and major genotypes. Asterisks represent the differential SSRs between two samples, and > 5 indicates that the length difference of differential SSRs between two samples is > 5 bp. SSRs with length differences less than 5 bp were hardly distinguished by electrophoresis. The name in the national standard represents the name of the differential SSR in the National Agricultural Standard of China No. NY/T 1433–3014. (XLSX 36 kb)

## Abbreviations

BB: Bacterial blight
bp: Base pair
CDS: Coding region
CE: Capillary electrophoresis
CRISPR/Cas9: Clustered regularly interspaced short palindromic repeats (CRISPR)/CRISPR-associated protein 9
CTAB: Hexadecyltrimethylammonium bromide
MAB: Marker-assisted backcrossing
NIL: Near-isogenic line
PCR: Polymerase chain reaction
PSA: Potato sugar agar
sgRNA: Small guide RNA
SNP: Single nucleotide polymorphism
SSR: Simple sequence repeat
WGS: Whole-genome sequencing
*Xoo*: *Xanthomonas oryzae* pv. *Oryzae*
