# Supplementary material for: Application of high-throughput amplicon sequencing-based SSR genotyping in genetic background screening
Source: BMC Genomics. 2019 Jun 3;20:444. doi: 10.1186/s12864-019-5800-4 (PMC6547574; doi:10.1186/s12864-019-5800-4)
Supplement: Supplementary file 1 — Figure S1. The sequencing figure shows the mutation patterns of the T0 transgenic lines 3 to 6. + 2, 2 bp insertion; − 10, 10 bp deletion; + 1, 1 bp insertion; − 3, 3 bp deletion. WT, Xa21 wild type; _ indicates inserted bases, and arrows indicate deletion start sites. Figure S2. Expression validation of the genes in which the differential SSRs were distributed. D62B was used as a reference sample, and the rice ubiquitin gene was used as an endogenous control. Figure S3. Development of DXB, DXT and Xa21m. O. longistaminata: Oryza longistaminata, Xa21 donor parent; IR24: Oryza sativa, recurrent parent. MAB: marker-assisted backcrossing. IRBB21, Xa21 donor parent for DXB. D62B, the recipient rice for DXB and DXT; DXB, Xa21 MAB-produced rice; DXT, Xa21 transgenic rice; Xa21m, CRISPR/Cas9-mediated Xa21 mutant rice. (PPTX 98 kb) [file 12864_2019_5800_MOESM1_ESM.pptx]

## Slide 1
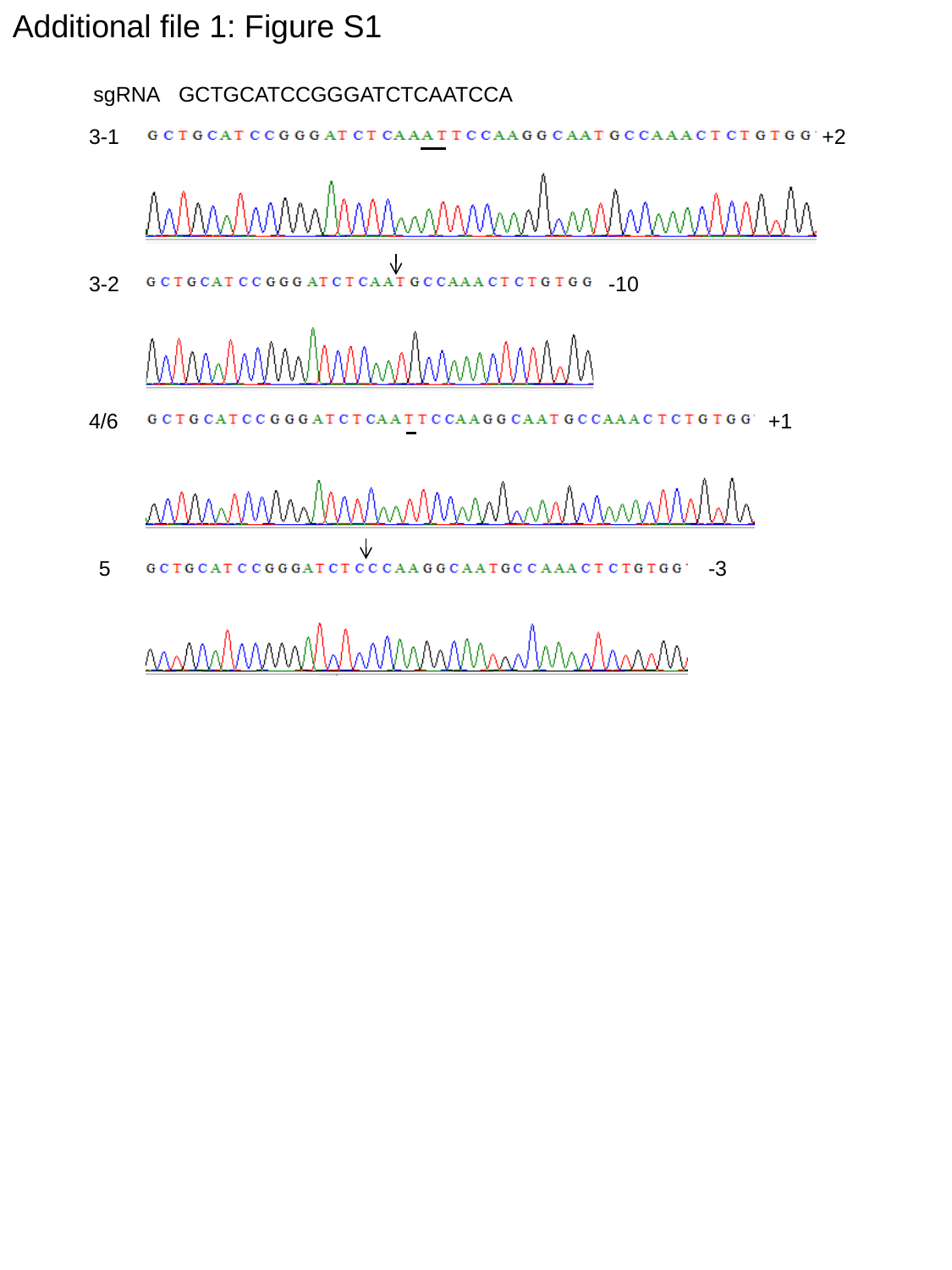

Additional file 1: Figure S1
sgRNA
GCTGCATCCGGGATCTCAATCCA
3-1
+2
-10
3-2
4/6
+1
5
-3

## Slide 2
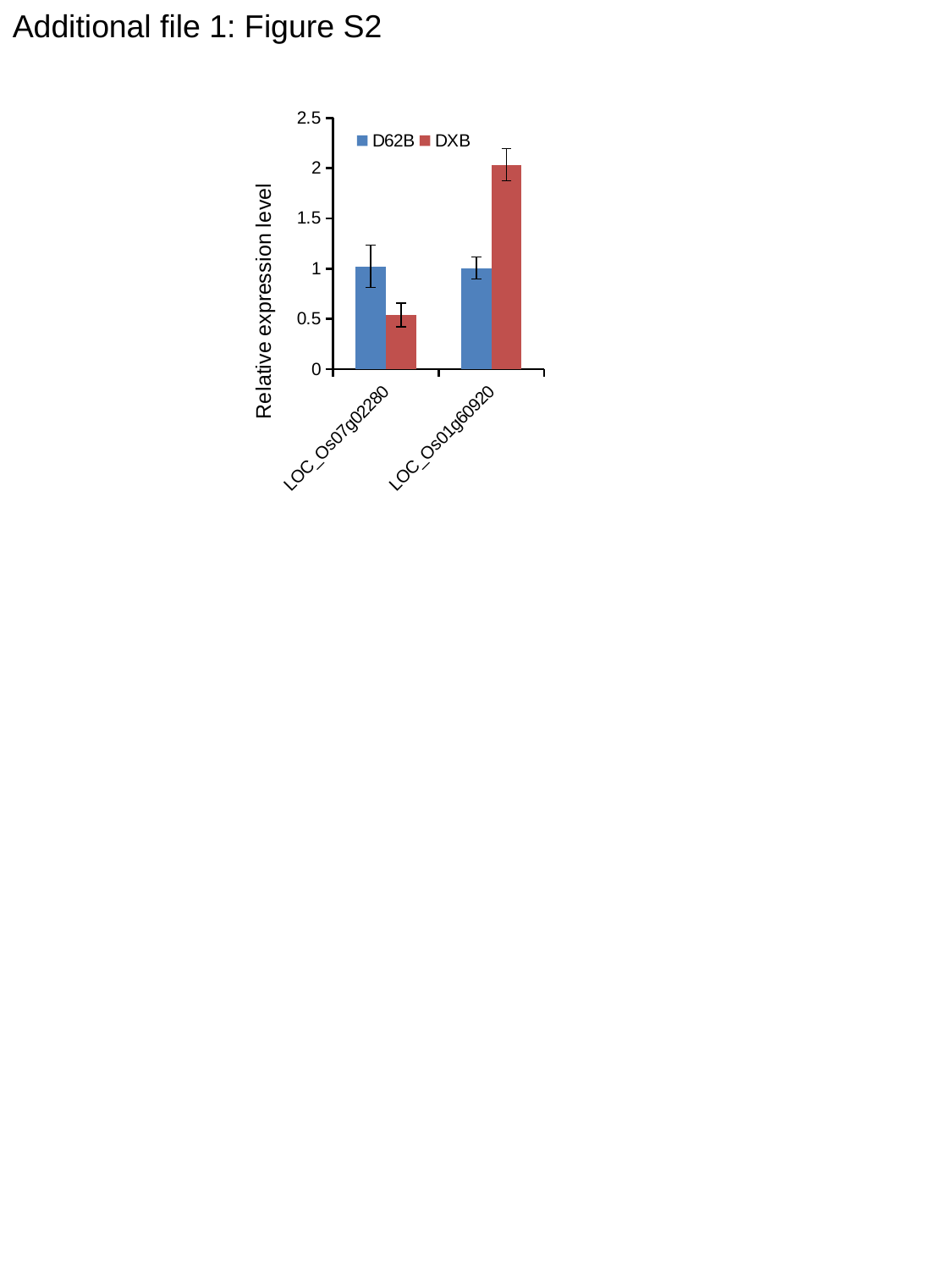

Additional file 1: Figure S2
### Chart
| Category | D62B | DXB |
|---|---|---|
| LOC_Os07g02280 | 1.0231488516858658 | 0.5393886650286434 |
| LOC_Os01g60920 | 1.0059132446320949 | 2.0341874058918523 |

## Slide 3
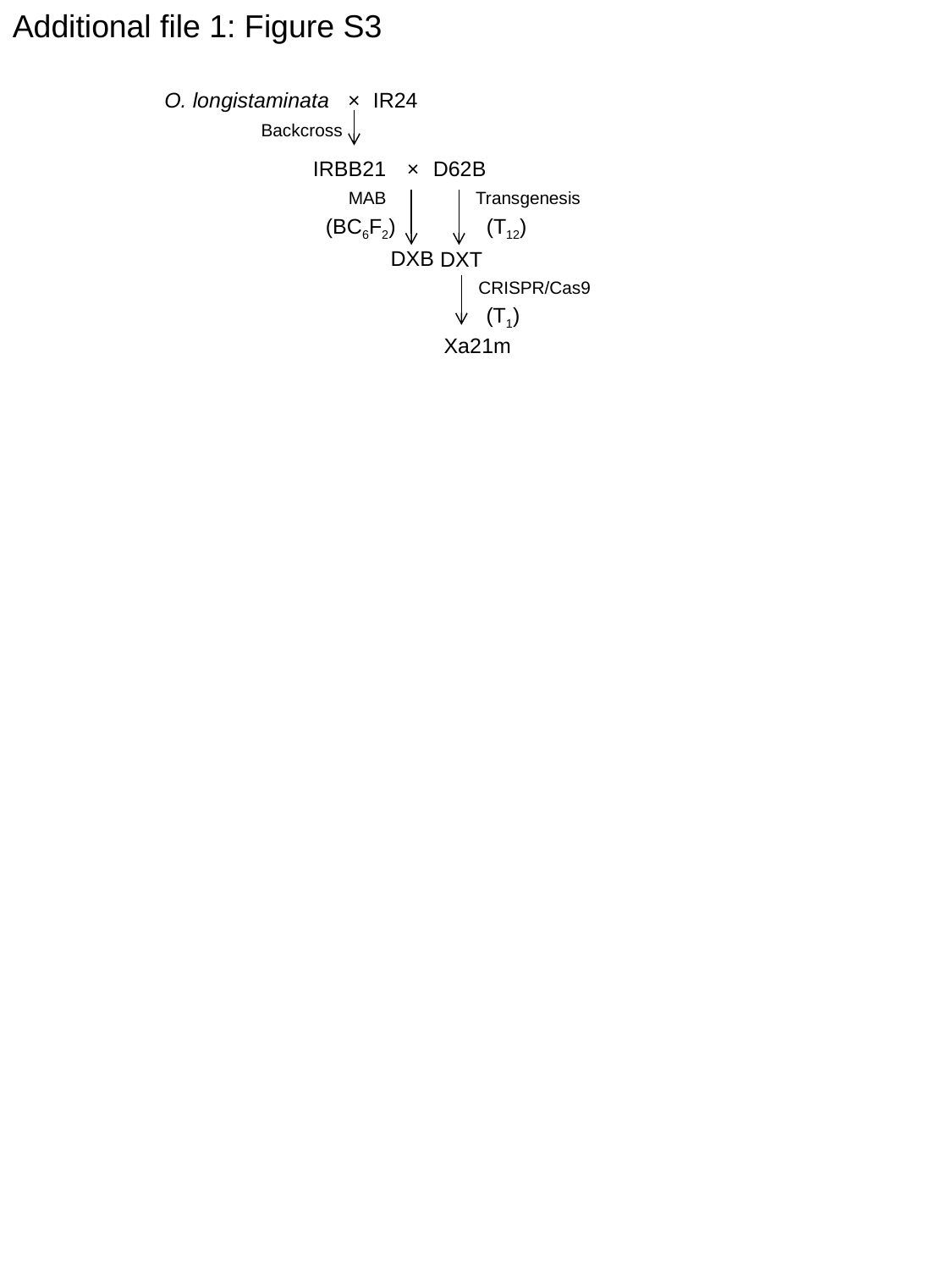

Additional file 1: Figure S3
O. longistaminata
×
IR24
 Backcross
IRBB21
×
D62B
MAB
Transgenesis
(BC6F2)
(T12)
DXB
DXT
CRISPR/Cas9
(T1)
Xa21m
